# Antiretroviral activity of the aminothiol WR1065 against Human Immunodeficiency virus (HIV-1) *in vitro *and Simian Immunodeficiency virus (SIV) *ex vivo*

**DOI:** 10.1186/1742-6405-6-24

**Published:** 2009-11-06

**Authors:** Miriam C Poirier, Ofelia A Olivero, Andrew W Hardy, Genoveffa Franchini, Jennifer P Borojerdi, Vernon E Walker, Dale M Walker, Gene M Shearer

**Affiliations:** 1 CDI Section, LCBG, CCR, National Cancer Institute, NIH, Bethesda, MD 20892, USA; 2 CMI Section, EIB, CCR, National Cancer Institute, NIH, Bethesda, MD 20892, USA; 3 AMRV, CCR, National Cancer Institute, NIH, Bethesda, MD 20892, USA; 4 University of Vermont, Burlington, VT, 05405, USA

## Abstract

**Background:**

WR1065 is the free-thiol metabolite of the cytoprotective aminothiol amifostine, which is used clinically at very high doses to protect patients against toxicity induced by radiation and chemotherapy. In an earlier study we briefly reported that the aminothiol WR1065 also inhibits HIV-1 replication in phytohemagglutinin (PHA)-stimulated human T-cell blasts (TCBs) infected in culture for 2 hr before WR1065 exposure. In this study we expanded the original observations to define the dose-response curve for that inhibition, and address the question of additive effects for the combination of WR1065 plus Zidovudine (AZT). Here we also explored the effect of WR1065 on SIV by examining TCBs taken from macaques with well-established infections several months with SIV.

**Results:**

TCBs from healthy human donors were infected for 2 hr with HIV-1, and viral replication (p24) was measured after 72 hr of incubation with or without WR1065, AZT, or both drugs. HIV-1 replication, in HIV-1-infected human TCBs, was inhibited by 50% at 13 μM WR1065, a dose at which 80% of the cells were viable. Cell cycle parameters were the same or equivalent at 0, 9.5 and 18.7 μM WR1065, showing no drug-related toxicity. Combination of AZT with WR1065 showed that AZT retained antiretroviral potency in the presence of WR1065. Cultured CD8^+ ^T cell-depleted PHA-stimulated TCBs from *Macaca mulatta *monkeys chronically infected with SIV were incubated 17 days with WR1065, and viral replication (p27) and cell viability were determined. Complete inhibition (100%) of SIV replication (p27) was observed when TCBs from 3 monkeys were incubated for 17 days with 18.7 μM WR1065. A lower dose, 9.5 μM WR1065, completely inhibited SIV replication in 2 of the 3 monkeys, but cells from the third macaque, with the highest viral titer, only responded at the high WR1065 dose.

**Conclusion:**

The study demonstrates that WR1065 and the parent drug amifostine, the FDA-approved drug Ethyol, have antiretroviral activity. WR1065 was active against both an acute infection of HIV-1 and a chronic infection of SIV. The data suggest that the non-toxic drug amifostine may be a useful antiretroviral agent given either alone or in combination with other drugs as adjuvant therapy.

## Background

Highly Active Antiretroviral Therapy (HAART) has revolutionized the treatment of HIV-1 disease and is primarily responsible for substantial improvements in the survival of HIV-1-infected patients seen in the last decade. However, the search for development of novel antiretroviral agents is ongoing and is largely driven by issues relating to drug resistance, formulation of drug combinations, pharmacokinetic profiles and toxicity. For example, combinations of nucleoside reverse transcriptase inhibitors (NRTIs) widely used in adult disease and for the prevention of maternal-fetal HIV-1 transmission have been instrumental in prolonging the lives of adults and saving the lives of thousands of children [1-4]. However, concern regarding mitochondrial and other toxicities in adults [5,6] and in HIV-1-uninfected children exposed *in utero *[7-9] to antiretroviral drugs has underscored the importance of designing strategies to both complement current antiretroviral cocktails and attenuate their toxic properties.

Amifostine [H_2_N(CH_2_)_3_NH(CH_2_)_2_S(PO_3_H_2_)], the FDA-approved drug Ethyol  is an organic thiophosphate that is dephosphorylated *in vivo *to the reduced free thiol WR1065 [H_2_N-(CH_2_)_3_NH-(CH_2_)_2_SH]. Amifostine inhibits radiation-induced mutagenesis in human [10] and hamster [11] cell lines. WR1065 selectively protects normal tissues, but not tumors, against ionizing radiation damage and chemotherapeutic drug cytotoxicity [12-14]. This compound has multiple biological activities, including ability to: detoxify reactive metabolites of chemotherapeutic agents; scavenge free radicals; modulate apoptosis; alter gene expression; and up-regulate mitochondrial manganese-superoxide dismutase [12,15].

Other thiols [16-18], and an analog of WR1065 [19], were reported to have antiretroviral activity. In addition, we showed in a pilot study that WR1065, the active free thiol metabolite, inhibits HIV-1 replication [20]. The cell culture studies presented here, using HIV-1 and the Simian Immunodeficiency Virus (SIV), are important preliminary steps towards our ultimate goal of evaluating the clinical efficacy of amifostine as an antiretroviral, or adjuvant-antiretroviral and/or adjuvant agent. *In vitro *studies are limited to the use of WR1065 because cells typically lack the alkaline phosphatase that is required to activate amifostine. Here we present: 1) the dose-response relationship for WR1065 antiretroviral activity in HIV-1-infected human T-cell blasts (TCBs) in the absence and presence of AZT; and 2) the antiretroviral effects of WR1065 in cultured TCBs from macaques infected chronically (14 months) with SIV.

## Methods

### Drug exposure and evaluation of virus replication in human T-cell blasts (TCBs)

Fresh human peripheral blood mononuclear cells (PBMC, from the NIH Transfusion Center) were cultured in 250 ml flasks (2 × 10^6 ^cells/ml) for 48 hr in RPMI-1640 media (ATCC, Manassas, VA) containing 10% fetal bovine serum (Hyclone, Logan, UT), 1% penicillin/streptomycin/glutamine (Invitrogen, Gaithersburg, MD), 10 U/ml interleukin 2 (IL2, BD Biosciences, San Jose, CA) and 20 μg/ml phytohemagglutinin (PHA, Sigma, St. Louis, MO). After 48 hr, the cells were washed to remove PHA and the resulting PHA-stimulated T-cell blasts (human TCBs) were transferred to 96 well microtiter plates (0.5 × 10^6 ^cells/well), infected with HIV-1_BZ-167_(gift from S. Sharpe, New York University, New York, NY) at 170-200 50% tissue culture infectious dose/10^5 ^target cells for 2 hr, and subsequently incubated with 2.5-103.0 μM WR1065 (Chemical Carcinogen Reference Standard Repository, Kansas City, MO) and/or 0.002-0.117 μM AZT (Sigma-Aldrich Inc., St. Louis, MO) for 72 hr. Cells were then harvested and evaluated for HIV-1 replication by RETRO-TEK HIV-1 p24 Extended Range Elisa Kit (ZeptoMetrix, Buffalo, NY) or by HIV-1 p24 Antigen Capture Assay Kit (Biological Products Laboratory, FCRDC, Frederick, MD).

To compare the metabolite WR1065 with the parent compound amifostine, in one experiment 50.0 μM amifostine (Chemical Carcinogen Reference Standard Repository) was added. Due to the lack of alkaline phosphatase in cultured human cells, we pre-incubated the amifostine with alkaline phosphatase (Sigma-Aldrich Inc.), at 1 U per 100 μl of media containing 50 μM amifostine, to generate WR1065. In experiments designed to examine virus replication with the combination of AZT and WR1065, the standard curve for AZT included concentrations between 0 and 23.0 ηM and WR1065 was used at either 18.7 or 26.0 μM.

### Cell survival of human TCBs

Drug-induced cell viability at 72 hr was determined by Trypan blue exclusion [20,21] in human TCBs grown in a second 96-well microtiter plate, where cells were exposed to drugs in the absence of HIV-1 inoculation. Cells from triplicate wells were mixed with Trypan blue and counted twice by hemocytometer. Numbers of viable (unstained) cells were expressed as a percentage of total (stained plus unstained) cells.

To examine apoptosis as a measure of cell viability in human TCBs infected with HIV-1 and treated with drug, we assayed for Annexin V (as previously described [22]). Cells taken from the wells used for p24 protein analysis were subjected to flow cytometry for this analysis and sorted on the basis of Annexin V positivity (apoptotic) and negativity (non-apoptotic).

### Flow Cytometry for determination of cell cycle parameters in human TCBs cultured in the presence of WR1065

Flow cytometry was used to evaluate the integrity of cell cycle parameters in human TCBs exposed to 0, 9.5 and 18.7 μM WR1065 according to the protocol described above. Harvested cells were pelleted and washed with culture media without serum before they were fixed overnight in 1 ml of ice-cold 70% ethanol, pelleted by centrifugation and incubated with Ribonuclease A (Sigma-Aldrich Inc.) at room temperature for 20 min. Propidium iodide (20-50 μg/ml) (Molecular Probes, Eugene, OR) was added to each cell suspension and cells were kept in the dark at 4°C overnight. Cells were passed through a fluorescence activated flow cytometer (FACSCalibur, BD Biosciences, San Jose, CA) using the doublet discrimination module, and data were acquired using CellQuest (BD Biosciences) software. Cell cycle analysis was performed using ModFit software (Venty Software, Topsham, ME). Percentages of cells in G_0_-G_1_, S and G_2_-M phases were calculated directly by the software.

### Culture of SIV-infected macaque TCBs and exposure to WR1065

Blood used to prepare macaque PBMC was collected from *Macaca mulatta *monkeys (macaques) numbered M612, M642 and M674. The macaques, housed at Advanced BioScience Laboratories (ABL), Inc. (Rockville, MD), had been infected with SIV_Mac251 _for 14 months before these experiments were performed. The animals were maintained and treated under conditions approved by the Association for Assessment and Accreditation of Laboratory Animal Care, and all procedures were performed in accordance with humane principles for laboratory animal care. Protocols were reviewed and approved by the Institutional Animal Care and Use Committee of ABL, Inc.

Macaque PBMC (10^6 ^cells/ml), prepared from blood using Ficoll gradient centrifugation, were depleted of CD8^+ ^cells by magnetic bead separation using the CD8 Microbead Kit for non-human primates (Miltenyi, Auburn, CA). Briefly, whole PBMC were incubated with microbeads conjugated to an anti-CD8^+ ^antibody and then washed. Cells were resuspended in Dulbecco's phosphate buffered saline (DPBS, Invitrogen, Carlsbad, CA) supplemented with 5% bovine serum albumin (BSA) and 2 mM EDTA, and run through a magnetic column. The flow-through material contained PBMC depleted (>99%) of CD8^+ ^T-cells, which were then counted and cultured using the same media as for the human TCBs (above). Once in culture, PBMC were incubated for 48 hr in the presence of PHA to activate remaining T-cells, as described above for human TCBs. These cells, macaque CD8^+ ^T cell-depleted, PHA-stimulated macaque T-cell blasts (TCBs) were transferred to 48-well plates (500 μL media/well, 0.5 × 10^6 ^cells/well, 6 wells/macaque) and cultured for an additional 17 days in the presence of 0, 9.5 or 18.7 μM WR1065. The medium was changed twice weekly for a total of 4 times, and fresh WR1065 was added at each medium change. Cell survival was evaluated on days 10 and 17 using the Cell Titer 96^® ^Aqueous Non-Radioactive Cell Proliferation (MTS) Assay (Promega Corp., Madison, WI). SIV levels were assayed using the p27 Antigen Assay kit (Beckman Coulter, Fullerton, CA) on days 3, 7, 10, 14 and 17.

## Results

### Anti-HIV-1 activity and cytotoxicity of WR1065 in human TCBs

In HIV-1-infected human TCBs, the HIV-1 titers, determined in the absence of drug, ranged from 1,312 to 38,000 pg p24/ml (10,205 ± 2,367, mean ± SE, n = 19 experiments). The inter-experimental variability, likely a reflection of the variability of HIV-1 growth in cells from different individuals, was such that we chose to present "% Inhibition" in the graphs and tables to take advantage of the power of multiple experiments. We assayed for WR1065-induced inhibition of HIV-1 replication at three points on the dose-response curve in several replicate experiments. The HIV-1 inhibition data are shown in Table 1, where 26 and 52 μM WR1065 gave 65% and 89% inhibition of HIV-1, respectively. Parallel cell survival studies were performed using either Trypan blue exclusion in cells with drug but no virus, or Annexin V, an early marker of apoptosis, in the HIV-1-infected cells containing drug (Table 1). Because the Trypan blue assay showed extensive cell death at 52 and 103 μM WR1065, we chose to perform subsequent experiments at ≤ 26 μM WR1065. For the Annexin V assay, drug-exposed cultures ranged from 75% to 100% Annexin V-negative (non-apoptotic), with the majority of experiments showing 85-95% of the cells as Annexin-V negative (data not shown).

**Table 1:** Inhibition of HIV-1 replication in human TCBs by WR1065 and amifostine.

**Concentration (μM)**	**Number of experiments**	**% Inhibition of^a ^HIV-1 replication (mean ± SE)**	**% Viability^b ^no HIV-1 Infection (Trypan blue)**	**% Viability^c ^HIV-1 Infection (Annexin V)**
***WR1065***				
26	5	65.0 ± 7.5	77.8 ± 4.2	95.8 ± 0.5
52	7	88.7 ± 5.5	49.3 ± 3.8	92.5 ± 1.1
103	8	93.9 ± 3.1	36.8 ± 17.1	ND^d^
***Amifostine***				
50	4	75.4 ± 8.3	53.2 ± 9.6	ND^d^

^a ^Human TCBs were infected with HIV-1 for 2 hr before incubation for 72 hr with WR1065, or amifostine converted to WR-1965 by pre-incubation with alkaline phosphatase. 50% inhibition of virus replication was at 13 μM WR1065.

^b ^Cell viability (mean ± SE), in HIV-1-uninfected cells, as determined by Trypan blue exclusion.

^c ^Cell viability (mean ± range, n = 2 experiments), in HIV-1-infected cells, was determined by Annexin V.

^d^ND = not determined.

Table 1 also presents mean values for replicate experiments in which we exposed HIV-1-infected human TCBs to amifostine to compare the anti-HIV-1 activity of this compound with its active metabolite WR1065. Because cultured human cells lack alkaline phosphatase, we pre-incubated 50 μM amifostine with this enzyme for 30 minutes before adding the mixture to HIV-1-infected human TCBs to evaluate viral replication. The extent of HIV-1 inhibition and the fraction of cells surviving were similar to those observed in cells cultured with 52 μM WR1065 (Table 1), indicating that most of the amifostine had been converted to WR1065 and was available to inhibit virus replication.

**Figure 1:**
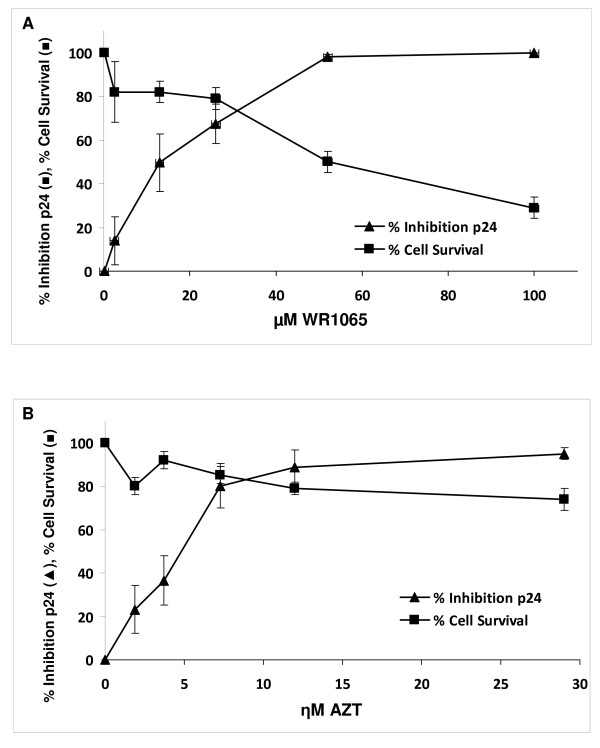
**(A) Concentration-dependent dose-response curve for % Inhibition (mean ± SE, n = 4 experiments) of HIV-1 replication in human TCBs incubated with 2.5, 13.0, 26.0, 51.5, 103.0 and 206.0 μM WR1065 for 72 hr and assayed by p24 ELISA (solid triangle)**. Cell survival (mean ± SE, n = 4 experiments) determined by Trypan blue exclusion (solid square). **(B) **Concentration dependent dose-response curve for HIV-1 replication in human TCBs incubated with 1.9, 3.7, 7.3, 11.7, 29.3 and 117.0 ηM AZT for 72 hr and assayed by p24 ELISA (solid triangle). Cell survival (mean ± SE, n = 4 experiments) determined by Trypan blue exclusion (solid square).

Complete dose-response curves for % inhibition of HIV-1 replication with WR1065, and TCB % survival determined by Trypan blue are plotted in Figure 1A (mean ± SE, n = 4 experiments). The concentration of WR1065 giving 50% inhibition of virus replication was 13 μM, and at this dose the TCBs were 80% viable by the Trypan blue. Also by Trypan blue, 50% cell survival was observed at 52 μM WR1065, yielding a therapeutic index of 0.25 for the cell culture studies. However, this relatively-poor *in vitro *therapeutic index is not relevant for the *in vivo *potential because the parent drug amifostine can be administered at very high doses with virtually no toxicity (see Discussion).

As an additional test of WR1065-induced toxicity, flow-cytometric analysis of cell cycle parameters, was performed using human TCBs grown in the presence of 0, 9.5 and 18.7 μM WR1065. Table 2 shows values for percentage of cells in S-phase, G_2_/M-phase and G_0_/G_1_ phase. We found that exposures of human TCBs to 9.5 and 18.7 μM WR1065 did not significantly alter the TCB cycling, as compared to unexposed cells, adding support to the notion that the TCBs did not sustain unacceptable toxicity at the doses chosen.

**Table 2:** WR1065 did not alter cell cycle parameters in HIV-1-uninfected human TCBs at non-toxic doses.^a^

**WR1065 (μM)**	**% Cells in S Phase**	**% Cells in G_2_/M Phase**	**% of Cells in G_0_/G_1_ Phase**
0	5.3 ± 1.6	1.3 ± 0.6	93.8 ± 2.2
9.5	6.2 ± 2.6	1.3 ± 0.7	92.5 ± 3.2
18.7	8.3 ± 2.5	1.5 ± 0.5	90.5 ± 2.6

^a ^Fresh human TCBs were infected with HIV-1 for 2 hr before incubation for 72 hr with WR1065. Values shown are mean ± range (n = 2). Cell viability, as determined by Trypan blue exclusion was 84.4-87.4% (mean ± range, n = 2).

### Anti-HIV-1 activity of AZT, with and without WR1065, in human TCBs

Figure 1B shows inhibition of HIV-1-replication, and cell survival determined by Trypan blue, for AZT dose-response experiments (mean ± SE, n = 4 experiments). The figure shows 50% inhibition of virus replication at 5.0 ηM AZT, a dose that was associated with 90% cell survival.

**Figure 2:**
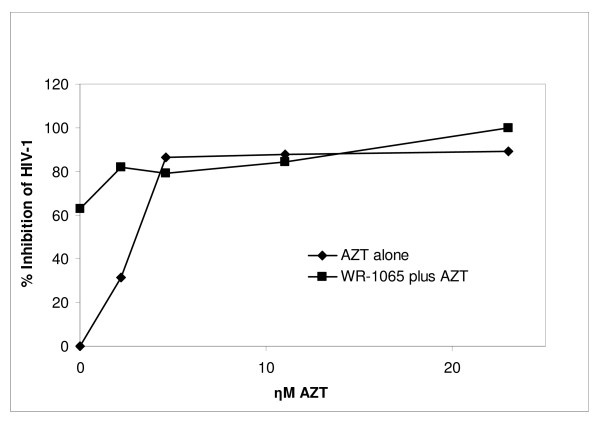
**Human TCB dose-response curves for: AZT alone (solid diamond, 0 -29.3 ηM); and the same doses of AZT with 18.7 μM WR1065 added to each dose (solid square)**. Note that WR1065 alone inhibited HIV-1 replication, and when WR1065 was added to 2.2 ηM AZT, the % inhibition of HIV-1 replication increased by 50%; high doses of AZT that completely inhibit virus replication were not informative.

We performed three experiments to examine inhibition of HIV-1 replication with the combination of AZT and WR1065 (Table 3). In each experiment, we compared two AZT dose-response curves, one with increasing doses of AZT alone, and a second with identical concentrations of AZT plus a constant amount of WR1065 added to each well. A representative experiment is shown in Figure 2, in which the increase in % inhibition of virus replication with both AZT and WR1065 is evident by comparing the curves with AZT alone (solid diamond) and AZT plus WR1065 (solid square). In this experiment (Experiment 3 from Table 3) the only dose of AZT that gave less-than-saturating inhibition of HIV-1 replication was 2.2 ηM. This AZT dose was informative because it did not saturate virus inhibition, allowing for further inhibition when WR1065 was added (see Table 3, right column). Due in part to interindividual differences in growth, HIV-1 infection capacity, and specific drug dose used, variability was such that the experiments could not be combined. However, the consistent increase in the % inhibition of HIV-1 replication with the addition of WR1065 to non-saturating doses of AZT (see Table 3, right column) suggests that WR1065 did not inhibit the antiretroviral activity of AZT. On the contrary, combination of WR1065 with AZT did increase the antiretroviral efficacy of AZT.

**Table 3:** Increase in AZT-induced % Inhibition of HIV-1 replication by the addition of non-toxic doses of WR1065^a^

**Expt. Number**	**% HIV-1 Inhibition WR1065 alone (Concentration)**	**% HIV-1 Inhibition AZT alone (Concentration)**	**% HIV-1 Inhibition AZT + WR1065**	**Increase in % inhibition HIV-1 with added WR1065 a**
1	65.2% (26.0 μM)	71.8% (1.9 ηM)	86.6%	**14.8%**
2	20.0% (26.0 μM)	38.8% (1.9 ηM)	61.4%	**29.9%**
3	67.4% (18.5 μM)	31.5% (2.2 ηM)	81.9%	**50.4%**

**a **In each experiment two identical AZT standard curves for inhibition of virus replication were compared; one curve had 1.9-23.0 ηM AZT alone and the second curve had 1.9-23.0 ηM AZT plus a constant non-toxic amount (see table) of WR1065 added to each AZT concentration. The only informative concentrations of AZT were the 1.9 and 2.2 ηM doses that alone gave % Inhibition values well below 80%. A representative set of curves (experiment 3) including all points is shown in Figure 2.

### Anti-SIV activity of WR1065 in TCBs from SIV-infected macaques

TCBs from three macaques, which had been chronically infected with SIV for 14 months, were used to test the effect of WR1065 on SIV replication *ex vivo*. At the time of blood collection, the animals (612, 642 and 674), had plasma titers of 0.10, 0.03 and 6.40 × 10^6 ^copies of SIV RNA/ml, and CD4 counts of 376, 635 and 547/ul, respectively. The PBMC were depleted of CD8^+ ^T cells, PHA stimulated, and either cultured for 20 days in the absence of WR1065, or cultured for 3 days before the addition of 0, 9.5 or 18.7 μM WR1065 to the medium, and then for an additional 17 days. The medium was changed twice weekly in both culture groups and fresh WR1065 was added at each medium change. Using the MTS assay, cell survival was measured on days 10 (data not shown) and 17 of this experiment (Table 4).

**Table 4:** Cell viability (%) in CD8^+ ^T cell-depleted TCBs, taken from 3 SIV-infected macaques, that were exposed to WR1065 for 17 days in culture

**WR1065 (μM)**	**Monkey Number**			**Mean ± SE**
		
	**612**	**642**	**674**	
		
0	100	100	100	100.0 ± 0.0
9.5	76	74	84	78.0 ± 3.0
18.7	73	63	75	70.3 ± 3.7

**Figure 3:**
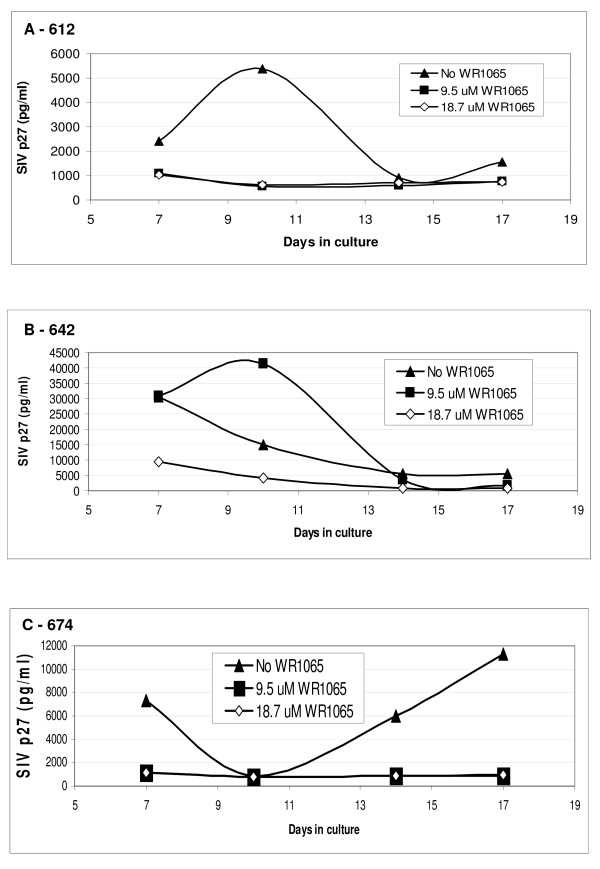
**SIV replication in macaque TCBs obtained from SIV-infected animals and cultured with 0 (solid triangle), 9.5 (solid square) or 18.7 (hollow diamond) μM WR1065 for 17 days**. SIV p27 values are shown for days 7, 10, 14 and 17 of culture for TCBs from macaques: (**A**) 612 (0.10 × 10^6 ^copies SIV/ml and 376 CD4 cells/ml); (**B**) 642 (0.03 × 10^6 ^copies SIV/ml and 635 CD4 cells/ml); and (**C**) 674 (6.40 × 10^6 ^copies of SIV/ml and 547 CD4 cells/ml).

Kinetic p27 data, generated in the cultures with and without WR1065, are illustrated in Figure 3. SIV replication by TCBs from macaque 612 (Figure 3A) cultured in the absence of WR1065 (solid triangle) peaked at day 10. In contrast, SIV replication was reduced approximately 5-fold, to background levels, in the two groups exposed to WR1065 (solid square, hollow diamond) (Figure 3A). The peak virus titer in the macaque 612 TCBs (5200 pg SIV/ml at 10 days) was the lowest of those examined. In TCBs from macaque 642 (Figure 3B), at 7 days, the SIV p27 levels in groups exposed to 0 (solid triangle) and 9.5 (solid square) μM WR1065 were measurable, and only in cells exposed to 18.7 μM WR1065 (hollow diamond) was the SIV titer lowered to background levels. At 7 days, TCBs cultured from macaque 642 had a virus titer of 30,000 pg SIV/ml (Figure 3B), which was much higher than the SIV titers for the other two macaques. Perhaps because of this, an antiviral effect was observed only at the 18.7 μM WR1065 dose in TCBs from macaque 642. In untreated TCBs from macaque 674 (Figure 3C), the virus titer (solid triangle) showed two viral peaks, one at day 7, followed by a decline at day 10, and a second increase for the remainder of the experiment. WR1065-exposed cells (solid square, hollow diamond) from this animal had baseline SIV levels throughout the 17 day culture period, indicating a persistent WR1065-induced inhibition of SIV replication. This inhibition was observed irrespective of fluctuations in the SIV replication pattern found in untreated cultures from different animals.

In summary, these *ex vivo *experiments performed using macaque TCBs obtained from three chronically-infected macaques demonstrate that WR1065 effectively inhibited production of SIV p27 throughout the 17-day culture period. Furthermore, our finding that 18.7 μM WR1065 was required to inhibit SIV replication in cultures with the highest SIV levels (Figure 3B) suggests that the inhibition of SIV replication is dose dependent.

## Discussion

These experiments demonstrate that WR1065 is effective in significantly reducing HIV-1 replication in cultured human TCBs infected with HIV-1 for 2 hr prior to treatment, and in macaque TCBs cultured from SIV-infected macaques for 17 days with the addition of WR1065. Taken together, these studies show inhibition of replication of two distinct retroviruses in TCBs from two different primate species. The data suggest that the parent drug, amifostine, which is non-toxic when used at very high doses *in vivo*, may have clinical utility. In addition, in combination studies using both AZT and WR1065 in human TCBs, we found that addition of WR1065 to a non-saturating dose of AZT resulted in more effective inhibition of HIV-1 replication than was observed with AZT alone, suggesting that amifostine might also be useful as supplementary or adjuvant therapy.

In a previous manuscript [20] we reported three pilot experiments using HIV-1, AZT and WR1065. The WR1065 doses used for those studies were very high (up to 1000 μM), and in only one of the three experiments did the dose range extend below 100 μM WR1065. Therefore, more information was required to determine the feasibility of initiating studies in primates. The experiments presented in this manuscript are essential because they define the dose-response parameters and show consistency in HIV-1 inhibition for >20 experiments. In addition, in this study the cytotoxicity was carefully defined in cell cycle and other experiments that were not performed previously. Finally, if amifostine is to be evaluated for use in humans it is important to show evidence of antiviral efficacy in SIV-infected macaques, and the *in vitro *studies presented here are a necessary a first step in the process.

Whereas amifostine has little or no toxicity in the clinic, WR1065 was cytotoxic in our cell cultures. This may have occurred partially as a result of the formation of WR1065 disulfide metabolites and other compounds. In long-term experiments this cytotoxicity can be prevented by the addition of aminoguanidine to the culture media [23]. However, because of the short duration of our human TCB studies we chose not to use aminoguanidine, and we lowered the WR1065 dose to ≤ 26 μM to obtain acceptable cell survival. Whereas the role of aminothiol oxidative metabolites may be critical for the interpretation of the cell culture studies, toxic metabolites do not appear to be an issue *in vivo *when amifostine is given. Additional experiments will be required to determine the *in vivo *efficacy of this drug.

The experiments in which AZT and WR1065 were given together were designed to investigate whether the antiviral efficacy of AZT might be inhibited in the presence of WR1065. The four experiments presented in Table 3 all showed that AZT was active in the presence of WR1065. In addition they suggested that there might be synergism in antiretroviral capacity when the drugs were combined, because for the informative doses, the AZT % Inhibition of HIV-1 replication was increased when WR1065 was added. This is an intriguing pilot finding, which requires much more detailed experimentation and statistical analysis for confirmation.

Amifostine, when dephosphorylated to WR1065, has cytoprotective activity that appears to be related both to the free thiol group and to the disulfide formed by interaction of the two WR1065 free thiol groups [13]. These aminothiol metabolites compete with polyamines to alter gene expression, stabilize DNA by electrostatic intercalation [12], act as free radical scavengers by binding to NFκB and p53 [24,25], thereby increasing transactivation of downstream genes, including manganese superoxide dismutase (MnSOD)[15]. WR1065 inhibits the catalytic site of Topoisomerase II [15] and up-regulates p21 [26,27]. Both of these genes are involved in cell cycle arrest and are relevant to the finding that WR1065-induced cytoprotection requires an intact and functioning DNA repair mechanism [12].

Amifostine is used at high doses to protect against the lethality of radiotherapy and chemotherapy in adults [28], and in pediatric oncology [29-31]. The recommended daily amifostine dose is 910 mg/M^2^, but higher doses are tolerated, and up to 2700 mg/M^2 ^has been used in children [32-34]. Pharmacokinetic studies, performed in humans and in monkeys [30,33,34], showed that administration of amifostine is followed by rapid dephosphorylation to WR1065, slower elimination of WR1065, and formation of various longer-lived metabolites. In one pharmacokinetic study, in children given 825 mg amifostine/M^2^, the peak concentration of WR1065 in whole blood, plasma and blood cells was 75, 85 and 83 μM, respectively [30]. In cynomolgus monkeys given subcutaneous amifostine at 260 mg/M^2^, the WR1065 peak plasma concentration was 104 μM [33]. In addition, bioavailability after oral administration yielded metabolites that persisted in the plasma for several hours [34]. The ability to achieve plasma and *in vivo *intracellular WR1065 levels in the range of 100 μM suggests that it may be possible to dose HIV-1 infected patients with amifostine levels that will sustain antiretroviral activity using FDA-recommended doses of drug. If amifostine is shown to be an effective clinical antiretroviral agent, it may be useful in patients who have developed resistance to conventional antiretroviral therapy, or as prophylaxis in HIV-1-uninfected health care workers who have been occupationally-exposed to HIV-1.

The mechanism(s) that may contribute to the antiretroviral efficacy of these drugs are still largely a matter of conjecture. One possible explanation comes from the importance of thiol-disulfide exchange in fusion of the HIV-1 envelope with host cell membrane, a process facilitated by protein disulfide isomerase [35,36]. Inhibitors of this enzyme prevent the establishment of virus infection. Also, retroviral inactivation has been accomplished using oxidizing agents that react with cysteine thiols in the zinc finger motifs of the retroviral nucleocapsid proteins [37,38]. The organic thiophosphate WR-151327, a methylated derivative of amifostine, inhibited HIV-1 reverse transcriptase activity and prevented the production of viral protein synthesis in a promonocytic cell line chronically-infected with HIV-1[19]. Inhibition of viral replication was maximal at 15 mM, a dose which exhibited no cytotoxicity for up to 7 days in culture. Several mechanisms, including modulation of glutathione, and NFκB-dependent and -independent pathways, were speculated to contribute to the observed inhibition of virus replication, and it is possible that those mechanisms may be relevant to our experiments with WR1065[19].

## Conclusion

The present study expands our original observation [23] that WR1065 inhibits the replication of HIV-1, by establishing dose-response curves for WR1065 and AZT alone, and showing that AZT has antiretroviral activity in the presence of WR1065. Furthermore, in this study we examined the *in situ *effect of WR1065 in a second primate species infected with an immunodeficiency virus inducing AIDS-like symptoms, and demonstrated that WR1065 inhibits SIV replication in TCBs activated from macaques infected for 14 months with SIV. These studies do not elucidate the underlying mechanisms of antiretroviral efficacy, but they are consistent with previous reports of HIV-1 and SIV replication inhibition induced by exposure of cultured cells to thiol-disrupting agents, and they may lead to useful supplementary and/or complementary clinical approaches for the management of HIV-1. Amifostine may have promise as an adjuvant antiretroviral agent because: it is non-toxic in humans and can be used at very high doses; human plasma levels can reach 50-100 μM, concentrations shown in culture to inhibit viral replication; it is an anti-mutagen and not likely to exhibit typical patterns of antiretroviral drug resistance involving mutagenesis; and, structurally the molecule is reasonably simple allowing for relatively inexpensive chemical synthesis.

## Abbreviations

AZT: Zidovudine; 3TC: Lamivudine; HAART: Highly active antiretroviral therapy; HIV-1: human immunodeficiency virus 1; IL2: interleukin 2; mnSOD: manganese superoxide dismutase; mtDNA: mitochondrial DNA; NRTI: nucleoside reverse transcriptase inhibitor; PHA: phytohemagglutinin; PBMC: peripheral blood mononuclear cells; human TCBs: PHA-stimulated T-cell blasts prepared from uninfected human PBMC; monkey TCBs: CD8^+ ^depleted, PHA-stimulated T-cell blasts prepared from PBMC taken from macaques infected with SIV for 14 months; SIV: simian immunodeficiency virus; WR2721: H_2_N(CH_2_)_3_NH(CH_2_)_2_S(PO_3_H_2_): amifostine or Ethyol; WR1065: H_2_N-(CH_2_)_3_NH-(CH_2_)_2_SH.

## Competing interests

The authors declare that they have no competing interests.

## Authors' contributions

DMW and VEW had the original idea for the use of WR1065 to attenuate the toxicity of nucleoside reverse transcriptase inhibitors, and from the beginning this was a collaboration with GMS who contributed labs with P3 containment where HIV-1 could be used. DMW and VEW provided essential information regarding the stability of WR1065 in culture, and funding to share the cost of the amifostine synthesis. MCP wrote the protocols, calculated the data, prepared the graphs and tables and wrote the paper. The actual experiments were performed in the laboratories of MCP and GMS using systems developed by GMS. GMS also provided critical conceptual input. OAO, JB, and AWH grew and treated the cells and performed the cytotoxicity assays and immunoassays for virus titer. OAO provided important conceptual input regarding the cytotoxicity assays. GF provided the monkey cells and was involved in the conceptual design of the SIV experiments. All authors read and approved the final manuscript.

## References

[B1] Wilson LE, Gallant JE (2009). HIV/AIDS: the management of treatment-experienced HIV-infected patients: new drugs and drug combinations. Clin Infect Dis.

[B2] Stek AM (2009). Antiretroviral medications during pregnancy for therapy or prophylaxis. Curr HIV/AIDS Rep.

[B3] Richman DD, Margolis DM, Delaney M, Greene WC, Hazuda D, Pomerantz RJ (2009). The challenge of finding a cure for HIV infection. Science.

[B4] Hammer SM, Eron JJ, Reiss P, Schooley RT, Thompson MA, Walmsley S (2008). Antiretroviral treatment of adult HIV infection: 2008 recommendations of the International AIDS Society-USA panel. JAMA.

[B5] Chiao SK, Romero DL, Johnson DE (2009). Current HIV therapeutics: mechanistic and chemical determinants of toxicity. Curr Opin Drug Discov Devel.

[B6] Calmy A, Hirschel B, Cooper DA, Carr A (2009). A new era of antiretroviral drug toxicity. Antivir Ther.

[B7] Barret B, Tardieu M, Rustin P, Lacroix C, Chabrol B, Desguerre I (2003). Persistent mitochondrial dysfunction in HIV-1-exposed but uninfected infants: clinical screening in a large prospective cohort. AIDS.

[B8] Brogly SB, Ylitalo N, Mofenson LM, Oleske J, van Dyke R, Crain MJ (2007). In utero nucleoside reverse transcriptase inhibitor exposure and signs of possible mitochondrial dysfunction in HIV-uninfected children. AIDS.

[B9] Foster C, Lyall H (2008). HIV and mitochondrial toxicity in children. J Antimicrob Chemother.

[B10] Grdina DJ, Dale P, Weichselbaum R (1992). Protection against AZT-induced mutagenesis at the *HGPRT *locus in a human cell line by WR-151326. Int J Radiation Oncol Biol Phys.

[B11] Grdina DJ, Nagy B, Hill CK, Wells RL, Peraino C (1985). The radioprotector WR1065 reduces radiation-induced mutations at the hypoxanthine-guanine phosphoribosyl transferase locus in V79 cells. Carcinogenesis.

[B12] Grdina DJ, Kataoka Y, Murley JS (2000). Amifostine: mechanisms of action underlying cytoprotection and chemoprevention. Drug Metabol Drug Interact.

[B13] Grdina DJ, Shigematsu N, Dale P, Newton GL, Aguilera JA, Fahey RC (1995). Thiol and disulfide metabolites of the radiation protector and potential chemopreventive agent WR-2721 are linked to both its anti-cytotoxic and anti-mutagenic mechanisms of action. Carcinogenesis.

[B14] Grdina DJ, Murley JS, Kataoka Y, Epperly W (2002). Relationships between cytoprotection and mutation prevention by WR-1065. Mil Med.

[B15] Kataoka Y, Murley JS, Khodarev NN, Weichselbaum RR, Grdina DJ (2002). Activation of the nuclear transcription factor kappaB (NFkappaB) and differential gene expression in U87 glioma cells after exposure to the cytoprotector amifostine. Int J Radiat Oncol Biol Phys.

[B16] Kalebic T, Kinter A, Poli G, Anderson ME, Meister A, Fauci AS (1991). Suppression of human immunodeficiency virus expression in chronically infected monocytic cells by glutathione, glutathione ester, and N-acetylcysteine. Proc Natl Acad Sci USA.

[B17] Simon G, Moog C, Obert G (1994). Effects of glutathione precursors on human immunodeficiency virus replication. Chem Biol Interact.

[B18] Ho WZ, Douglas SD (1992). Glutathione and N-acetylcysteine suppression of human immunodeficiency virus replication in human monocyte/macrophages in vitro. AIDS Res Hum Retroviruses.

[B19] Kalebic T, Schein PS (1994). Organic thiophosphate WR-151327 suppresses expression of HIV in chronically infected cells. AIDS Res Hum Retroviruses.

[B20] Walker DM, Kajon AE, Torres SM, Carter MM, McCash CL, Swenberg JA (2009). WR1065 mitigates AZT-ddI-induced mutagenesis and inhibits viral replication. Environ Mol Mutagen.

[B21] Freshney R (1987). Culture of Animal Cells: A Manual of Basic Technique.

[B22] Herbeuval JP, Grivel JC, Boasso A, Hardy AW, Chougnet C, Dolan MJ (2005). CD4+ T-cell death induced by infectious and noninfectious HIV-1: role of type 1 interferon-dependent, TRAIL/DR5-mediated apoptosis. Blood.

[B23] Walker DM, Torres SM, Kajon AE, Carter MM, McCash CL, Swenberg JA (2009). In vitro pilot studies of WR1065-mediated activity against NRTI-induced cytotoxicty and mutagenesis, and antiviral efficacy against HIV-1, influenza A and B viruses, and adenoviruses. Environmental and Molecular Mutagenesis.

[B24] Shen H, Chen ZJ, Zilfou JT, Hopper E, Murphy M, Tew KD (2001). Binding of the aminothiol WR-1065 to transcription factors influences cellular response to anticancer drugs. J Pharmacol Exp Ther.

[B25] Pluquet O, North S, Bhoumik A, Dimas K, Ronai Z, Hainaut P (2003). The cytoprotective aminothiol WR1065 activates p53 through a non-genotoxic signaling pathway involving c-Jun N-terminal kinase. J Biol Chem.

[B26] Snyder RD, Grdina DJ (2000). Further evidence that the radioprotective aminothiol, WR- catalytically inactivates mammalian topoisomerase II. Cancer Res.

[B27] Mann K, Hainaut P (2005). Aminothiol WR1065 induces differential gene expression in the presence of wild-type p53. Oncogene.

[B28] Koukourakis MI, Abatzoglou I, Sivridis L, Tsarkatsi M, Delidou H (2006). Individualization of the subcutaneous amifostine dose during hypofractionated/accelerated radiotherapy. Anticancer Res.

[B29] Stolarska M, Mlynarski W, Zalewska-Szewczyk B, Bodalski J (2006). Cytoprotective effect of amifostine in the treatment of childhood neoplastic diseases--a clinical study including the pharmacoeconomic analysis. Pharmacol Rep.

[B30] Souid AK, Fahey RC, Dubowy RL, Newton GL, Bernstein ML (1999). WR-2721 (amifostine) infusion in patients with Ewing's sarcoma receiving ifosfamide and cyclophosphamide with mesna: drug and thiol levels in plasma and blood cells, a Pediatric Oncology Group study. Cancer Chemother Pharmacol.

[B31] Anacak Y, Kamer S, Haydaroglu A (2007). Daily subcutaneous amifostine administration during irradiation of pediatric head and neck cancers. Pediatr Blood Cancer.

[B32] Adamson PC, Balis FM, Belasco JE, Lange B, Berg SL, Blaney SM (1995). A phase I trial of amifostine (WR-2721) and melphalan in children with refractory cancer. Cancer Res.

[B33] Bachy CM, Fazenbaker CA, Kifle G, McCarthy MP, Cassatt DR (2004). Tissue levels of WR-1065, the active metabolite of amifostine (Ethyol), are equivalent following intravenous or subcutaneous administration in cynomolgus monkeys. Oncology.

[B34] Mangold DJ, Huelle BK, Miller MA, Geary RS, Sanchez-Barona DO, Swynnerton NF (1990). Pharmacokinetics and disposition of WR-1065 in the rhesus monkey. Drug Metab Dispos.

[B35] Ryser HJ, Levy EM, Mandel R, DiSciullo GJ (1994). Inhibition of human immunodeficiency virus infection by agents that interfere with thiol-disulfide interchange upon virus-receptor interaction. Proc Natl Acad Sci USA.

[B36] Markovic I, Stantchev TS, Fields KH, Tiffany LJ, Tomic M, Weiss CD (2004). Thiol/disulfide exchange is a prerequisite for CXCR4-tropic HIV-1 envelope-mediated T-cell fusion during viral entry. Blood.

[B37] Arthur LO, Bess JW, Chertova EN, Rossio JL, Esser MT, Benveniste RE (1998). Chemical inactivation of retroviral infectivity by targeting nucleocapsid protein zinc fingers: a candidate SIV vaccine. AIDS Res Hum Retroviruses.

[B38] Rossio JL, Esser MT, Suryanarayana K, Schneider DK, Bess JW, Vasquez GM (1998). Inactivation of human immunodeficiency virus type 1 infectivity with preservation of conformational and functional integrity of virion surface proteins. J Virol.

